# When Cuts Cost More: Projected Fiscal Impact of Eliminating the AIDS Drug Assistance Program in 30 US States

**DOI:** 10.64898/2026.07.27.26359045

**Published:** 2026-07-31

**Authors:** Ryan M. Forster, Melissa Schnure, Ruchita Balasubramanian, Joyce L. Jones, Emily P. Hyle, Scott Batey, Keri N Althoff, Kelly Gebo, David W. Dowdy, Maunank Shah, Anthony T. Fojo, Parastu Kasaie

## Abstract

Across 30 US states and the District of Columbia, eliminating the AIDS Drug Assistance Program is projected to save $8.32 billion in direct costs while generating $14.89 billion in downstream HIV care costs attributable to excess incident infections from 2026-2035. Costs are projected to surpass savings within six years.

## Full Text Availability

The license terms selected by the author(s) for this preprint version do not permit archiving in PMC. The full text is available from the preprint server.

